# Authorship diversity in otolaryngology: a 9-year analysis of articles published in Journal of Otolaryngology—Head and Neck Surgery

**DOI:** 10.1186/s40463-023-00667-0

**Published:** 2023-09-06

**Authors:** Keshinisuthan Kirubalingam, Agnieszka Dzioba, Yvonne Chan, M. Elise Graham

**Affiliations:** 1 Department of Otolaryngology–Head and Neck Surgery, Children’s Hospital, London Health Sciences Centre, Victoria Hospital, Schulich School of Medicine and Dentistry, Western University, 800 Commissioners Road E, Box 5010, London, ON N6A 5W9 Canada; 2 Department of Otolaryngology–Head and Neck Surgery, St Michael’s Hospital, University of Toronto, Toronto, ON Canada

## Introduction

Although there are more women in medicine now than ever before, gender parity is still not reflected in leadership positions within academic medicine [1, 2]. Article authorship is an important distinction in academic medicine and often used as a surrogate for scholarly productivity. While US studies show increasing female-authored articles in Otolaryngology-Head and Neck Surgery (OHNS), there is a lack of Canadian research on gender diversity in authorship [3, 4]. The goal of this study was to examine trends in female authorship within the Canadian Journal of Otolaryngology—Head and Neck Surgery.

## Methods

All research articles published online in the journal were analyzed from 2013 to the end of 2021. The genders of first, middle and senior authors were recorded along with the article type, article category and institutional affiliations. The total number of female authors including unique authors, along with the number of first and senior female authors specifically, were quantified. Gender was identified through name searches and online sources. Proportions of same-gender first and senior authors were calculated. Cochran-Armitage trend tests were used to assess the change in proportion over time between years and groups. Chi-square tests of gender differences were used to assess gender representation by article category and type. The study used publicly available, de-identified data and was exempt from institutional ethics board review.

## Results

Of the 604 publications, 75 were excluded as they were corrections, addendums, reviewer acknowledgements, or author gender could not be determined. 529 articles with 3058 total authors were included with 727 (23.8%) female authors and 2331 (76.2%) male authors (*P* < 0.001). Of the 529 first authors, 157 (29.7%) were female and 372 (70.3%) were male (*P* < 0.001). Of the 521 senior authors, 74 (14.2%) were female and 447 (85.8%) were male (*P* < 0.001) (Table 1).

**Table 1:** Proportion of gender by authorship

Authorship	Female authors		Male authors		*P* value
	Total	Unique	Total	Unique	
All authors	727 (23.8%)	523 (29.4%)	2331 (76.2%)	1253 (70.6%)	< 0.001
First authors	157 (29.7%)	128 (31.6%)	372 (70.3%)	277 (68.4%)	< 0.001
Senior authors	74 (14.2%)	51 (16.7%)	447 (85.8%)	254 (83.3%)	< 0.001

The proportion of female first authors (25.4% in 2013 to 35.7% in 2021; *P* = 0.298) and senior authors (6.8% in 2013 to 10.7% in 2021; *P* = 0.605) did not increase significantly over time. The proportion of articles with same gender first and senior author by year revealed a non-significant decrease in male first author to male senior author collaborations (72.9% in 2013 to 57.1% in 2021; *P* = 0.052). There was a significant difference in gender representation in senior authorship by article category, notably in facial plastics and rhinology (*P* = 0.005) (Table 2).

**Table 2:** Authorship gender representation by article category

Article category	First authors *P* = 0.100		Senior authors *P* = 0.005	
	Female (%)	Male (%)	Female (%)	Male (%)
Education	17 (50)	17 (50)	10 (29.4)	24 (70.6)
Facial plastics	4 (26.7)	11 (73.3)	0 (0)	15 (100)
General OHNS	11 (20.4)	43 (79.6)	10 (19.2)	42 (80.8)
Head and neck	59 (28.4)	149 (71.6)	25 (12)	183 (88)
Laryngology	13 (39.4)	20 (60.6)	9 (28.1)	23 (71.9)
Otology	24 (25.5)	70 (74.5)	10 (10.9)	82 (89.1)
Pediatric OHNS	8 (40)	12 (60)	4 (21.1)	15 (78.9)
Rhinology	21 (30.4)	48 (69.6)	5 (7.5)	61 (92.5)

## Discussion

The present study highlights the trends in female authorship representation within the Canadian *Journal of Otolaryngology Head and Neck Surgery*. These findings suggest that female OHNS surgeons are participating in research overall at similar rates at which they are represented in the Canadian OHNS community [5]. Despite the upward trends in research participation, our findings suggest that at the senior authorship level, women continue to be under-represented. It is worth noting that female representation at the staff and leadership level adds an important context. A recent cross-sectional Canadian study identified that women are underrepresented in staff and senior academic roles, including both professorship and departmental leadership roles [6].

Although there has been an increase in the proportion of female authors in the Canadian Journal, our findings suggest that there is no significant change in female authorship representation throughout the study period. By leveraging quantitative findings, future studies should identify barriers to female advancement within the field and encourage actionable efforts such as promotion of women in academia and diversification of research opportunities. In doing so, we will be able to dismantle the hurdles and encourage sponsorship of female OHNS surgeons in their academic contributions to the field.

## Data Availability

Data is available upon reasonable request.
